# Sustained invisibility through crowding and continuous flash suppression: a comparative review

**DOI:** 10.3389/fpsyg.2014.00475

**Published:** 2014-05-27

**Authors:** Nathan Faivre, Vincent Berthet, Sid Kouider

**Affiliations:** ^1^Division of Biology, Computation and Neural Systems, California Institute of TechnologyPasadena, CA, USA; ^2^Laboratory of Cognitive Neuroscience, Brain Mind Institute, School of Life Sciences, École Polytechnique Fédérale de LausanneLausanne, Switzerland; ^3^University of LorraineInterPsy, Nancy, France; ^4^Laboratoire de Sciences Cognitives et Psycholinguistique, EHESS/CNRS/ENS-DECParis, France

**Keywords:** visual crowding, continuous flash suppression, unconscious processing, awareness, consciousness

## Abstract

The study of non-conscious vision benefits from several alternative methods that allow the suppression of an image from awareness. Here, we present and compare two of them that are particularly well-suited for creating sustained periods of invisibility, namely visual crowding and continuous flash suppression (CFS). In visual crowding, a peripheral image surrounded by similar flankers becomes impossible to discriminate. In CFS, an image presented to one eye becomes impossible to detect when rapidly changing patterns are presented to the other eye. After discussing the experimental specificities of each method, we give a comparative overview of the main empirical results derived from them, from the mere analysis of low-level features to the extraction of semantic contents. We conclude by proposing practical guidelines and future directions to obtain more quantitative and systematic measures of non-conscious processes under prolonged stimulation.

## INTRODUCTION

For long, the most prevalent method used to disrupt visual awareness was masking, which consists in presenting a stimulus very briefly, in temporal contiguity with noise patterns (Breitmeyer and Ogmen, 2006). Visual masking has been extremely fruitful in describing both the architecture of the visual system and the properties of non-conscious vision (Kouider and Dehaene, 2007). Yet, as masked stimuli become visible when presented for longer than a few 10s of milliseconds, masking is not well suited for the investigation of cognitive functions requiring sustained stimulation such as motion processing, perceptual learning, sequential learning, visual search, temporal integration, etc.

In this review, we present and compare two techniques that, contrary to masking, allow for prolonged stimulation while maintaining a reliable control of awareness. The first method, visual crowding, makes a peripheral object surrounded by similar flankers impossible to discriminate, so that one cannot determine consciously some of its specific features like its angular orientation, shape, or color (Levi, 2008; Pelli and Tillman, 2008; Whitney and Levi, 2011). The second method, continuous flash suppression (CFS), renders an object presented to one eye undetectable when the other eye is flashed with a stream of rapidly changing patterns, so that one cannot determine consciously whether the stimulus is present or absent (Tsuchiya and Koch, 2005; for reviews see Lin and He, 2009; Sterzer et al., 2014). A major distinction between crowding and CFS is at the phenomenological level: while crowding prevents stimulus discriminability, CFS prevents stimulus detectability (although situations of partial awareness exist in CFS, see below). Hereafter, unless specified, “invisibility” refers to the absence of discrimination under crowding (i.e., one feature of interest is not consciously perceived, although the presence vs. absence of the stimulus is detected), and to the absence of detection under CFS (i.e., the presence vs. absence of one feature of interest or the whole stimulus is not detected). We focused on CFS and crowding as they constitute, as of today, the two most suited methods to study the temporal dynamics of conscious vs. non-conscious vision. Although other methods can induce long periods of invisibility, they are not suited for psychophysical procedures (e.g., inattentional blindness, which is effective for a few trials only), and they do not allow for a strict control of stimulus duration (e.g., binocular rivalry or bistable figures, in which visibility fluctuates erratically over time; see Kim and Blake, 2005 for a review). The phenomena of crowding and CFS are driven by specific properties of the visual system and constitute by themselves specific research questions. Only those that are relevant for the field of non-conscious vision will be covered here, as our primary goal is to offer a description of how these two methods contribute to the study of non-conscious perception, and by extension to our understanding of the mechanisms underlying consciousness. After discussing the specificities implied by both methods in terms of experimental design and procedure, we give a comparative overview of the main empirical results derived from each of them. In light of this reviewing work, we propose criteria for choosing one method over the other depending on the research question at hand. In addition, we diagnose three kinds of methodological limitations commonly found in empirical studies: the use of “all-or-none” experimental designs, the absence of methodological comparisons, and the lack of systematicity when estimating stimulus visibility. Accordingly, we propose tentative guidelines to overcome those limitations and to evaluate, in a more quantitative and systematic fashion, the nature of non-conscious vision.

## HOW CFS AND CROWDING ARE USED?

**FIGURE 1:**
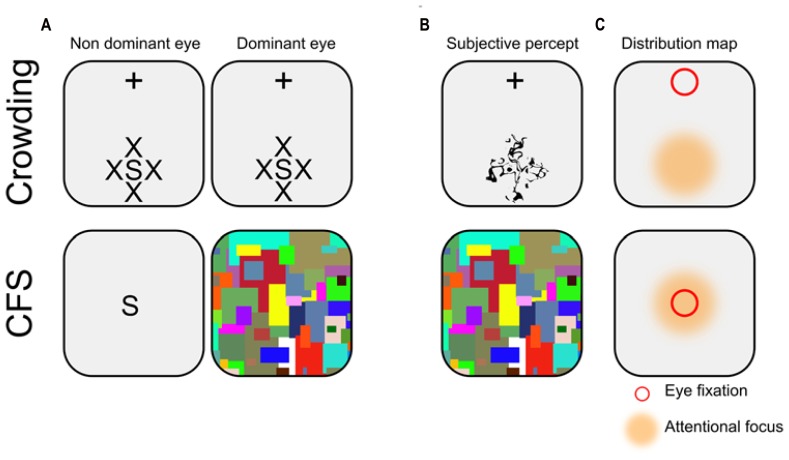
**Illustration of typical crowding and CFS displays.** **(A)** Schematic representation. In crowding, participants stare at a fixation cross (which here appears at the top of the screen), while a stimulus of interest (here the letter S) is presented in the periphery of the visual field, surrounded by similar flankers (here, the letters X). Note that the display is constant across eyes. In CFS, the stimulus of interest is usually presented foveally to the non-dominant eye, while the dominant eye receives a stream of rapidly changing pattern called mondrians. **(B)** Subjective percept. In crowding, observers report seeing a jumbled array of letters in the periphery (i.e., discrimination, but not detection is impeded). In CFS, observers typically report seeing only the mondrians (i.e., both discrimination and detection are impeded). **(C)** Distribution map. In crowding, eye fixation (depicted here by a red square) and attentional focus (depicted here by an orange area) are always dissociated (i.e., endogenous attention), while they are usually conjoint in CFS.

CFS and crowding are of great relevance to study non-conscious processes involving long-lasting stimuli. Although both methods can be used alternatively in this context, they are supported by different mechanisms, and are associated with distinct subjective experiences (see **Figure 1**). Contrary to crowding which is a natural phenomenon occurring during normal vision, CFS has been developed explicitly as a tool to study non-conscious processes. Therefore, the psychophysical properties of CFS are far less described than those of crowding. Hence, the following description is based on both published results and personal, undocumented observations which we hope summarize the general opinion of researchers in the field.

### HOW TO ARRANGE STIMULI?

As mentioned above, in CFS, the stimulus of interest is typically presented foveally and in isolation to the non-dominant eye, while the dominant eye is flashed with a series of salient patterns called mondrians and changing every 100 ms. In order for suppression to be reliable, the mondrians have to share a maximum of featural similarities with the stimulus of interest, notably in terms of shape, spatial frequency, or color (Hong and Blake, 2009; Zadbood et al., 2011; Yang and Blake, 2012). Crowding involves the binocular presentation of a peripheral stimulus surrounded by similar flankers. Importantly, crowding does not depend on stimulus size (Tripathy and Cavanagh, 2002), which allows for preventing awareness of relatively large stimuli (i.e., around 5° of visual angle; to our knowledge, no study has systematically investigated this aspect with CFS). Typically, the eccentricity between the center of the stimulus of interest and gaze location ranges from 5 to 20° of visual angle. According to an empirical law, the center-to-center spacing between the stimulus of interest and its flankers must be inferior to half the eccentricity in order for crowding to occur (Bouma, 1970). In addition, the degree of eccentricity and critical spacing needed to induce crowding varies depending on several factors such as the stimulus complexity (e.g., number and density of features, see Bernard and Chung, 2011), and the similarity between the stimulus of interest and its flankers (e.g., in terms of shape, see Kooi et al., 1994, or spatial frequency, see Chung et al., 2001), as well as their spatial arrangement (Livne and Sagi, 2007, 2010; Saarela et al., 2009; Manassi et al., 2012). Even though crowding applies to simple stimuli (van den Berg et al., 2007), one can notice that the level of crowding necessary to reach chance-level discrimination is usually obtained at smaller eccentricities for multi-feature symbols (e.g., typically 5° for Chinese pictographs, see Yeh et al., 2012) than for single feature stimuli (e.g., typically 20° for oriented lines, see Faivre and Kouider, 2011a)^1^. Accordingly, the disruption of awareness for such simple stimuli might involve not only crowding, but also unspecific mechanisms leading to signal loss at high eccentricities in the visual field (e.g., decrease of attentional amplification, decrease of photo-receptors in the peripheral retina, poor sensitivity in the high frequency domain etc.). Note however, that such peripheral limitations can be counter-balanced by scaling up stimuli, as the extent of crowding does not scale with stimulus size (Tripathy and Cavanagh, 2002). As opposed to crowding, the efficacy of CFS does not seem to correlate with stimulus complexity, which may qualify the latter as a preferred method for preventing awareness of simple, single feature stimuli like oriented lines, or color patches.

### HOW LONG CAN WE KEEP IT INVISIBLE?

The duration of stimulation is a determinant factor in experimental designs for two main reasons. First, long-lasting stimulation allows for studying the non-conscious processing of dynamic stimuli (e.g., motion, see below for empirical results). Second, it allows for manipulating stimulus onset asynchronies (SOA) independently of visibility, contrary to what happens during backward masking in which an increase of SOA is usually associated with an increase in visibility (Weisstein and Haber, 1965). Note that longer is not necessarily better, as increasing stimulus duration can have the consequence of inversing facilitatory priming effects into inhibitory habituation effects for stimuli rendered invisible, whether it is for crowding (Faivre and Kouider, 2011a) or for CFS (Barbot and Kouider, 2012; Faivre and Koch, 2014a).

How sustained is invisibility under crowding and CFS? Although no study investigated directly whether peripheral stimuli could escape crowding after relatively long exposures (i.e., minutes), previous studies suggest that there is no upper limit (e.g., Kooi et al., 1994). Thus, in principle, one might be able to prevent awareness for extended durations, as long as observers do not stare directly at the stimulus. This last aspect requires oculometric control, and notably the use of a gaze-contingent display, in which the stimulus of interest is replaced by a non-informative pattern as soon as the observer stops staring at the fixation cross. We called the experimental procedure fulfilling this condition gaze contingent crowding (GCC; Faivre and Kouider, 2011b; Kouider et al., 2011). Compared to crowding, oculometric control in CFS is far less developed, mostly due to the use of stereoscopes that are usually not compatible with eye-tracking (but see Rothkirch et al., 2012, for oculomotor correlates of non-conscious processing under CFS). No study investigated systematically the maximal duration of invisibility allowed by CFS. In their seminal study, Tsuchiya and Koch (2005) noted that in around 15% of trials “no part of the gray image was seen at all for the full 3-min trial.” In general though, stimuli are known to break suppression after several seconds of display. In fact, the time taken by a stimulus to break suppression is now commonly used as a dependent variable for inferring non-conscious processing [i.e., “breaking CFS,” see Jiang et al., 2007; and the review by Gayet et al. (2014) and Stein and Sterzer (2014) in the present volume].

### HOW IMPORTANT IS ATTENTION?

Although the links between attention and consciousness are intricate and complex (Lamme, 2003; Koch and Tsuchiya, 2007; Cohen et al., 2012), there is now a general consensus that both conscious and non-conscious processes are themselves influenced by attentional mechanisms. For our purpose here, attention seems to plays a dual role. First, attentional amplification is known to decrease the impact of crowding and thus to increase visibility (Yeshurun and Rashal, 2010; to our knowledge no direct investigation of the links between attention and the strength of suppression under CFS has been performed). In addition, even without awareness, non-conscious effects are of bigger amplitude when stimuli are attended vs. unattended, both in crowding (Montaser-Kouhsari and Rajimehr, 2005; Faivre and Kouider, 2011b) and in CFS (Kanai et al., 2006; Bahrami et al., 2008; Kaunitz et al., 2011; see also the Frontiers research topic dedicated to this issue by Tsuchiya and van Boxtel, 2013). In CFS, the location of attentional focus is usually superimposed to the location of eye fixation (but see Bahrami et al., 2007; Hesselmann and Malach, 2011; Yuval-Greenberg and Heeger, 2013 for notable exceptions). By contrast, in crowding, the location of attentional focus is never superimposed to the location of eye fixation (e.g., observers stare at a fixation point presented at the top the screen, while endogenously attending to a stimulus presented at the bottom of the screen). Thus, in order to maximize effects amplitudes, participants have to deploy their attentional focus away from where they are staring at.

### CAN WE CONTRAST VISIBLE AND INVISIBLE SITUATIONS AT CONSTANT STIMULATION?

One crucial condition to isolate the neural correlates of visual consciousness with a contrastive approach is to compare two states of consciousness while the stimulation remains constant (Baars, 1998). When this condition is fulfilled, observed differences between the two states of consciousness can hardly be argued to reflect confounds in terms of signal strength. This condition is well verified with ambiguous figures, binocular rivalry, or attentional blink (see Kim and Blake, 2005 for a review). Some efforts have been made to apply the same logic to visual masking: by reversing the order of masks and blanks, a stimulus is either visible when directly surrounded by blanks, or invisible when surrounded by masks (e.g., Dehaene et al., 2001). Although this method provided valuable results, one can still deplore that it only fulfills the criterion of constant stimulation at a global scale (i.e., when integrating the signal over the whole trial duration), but not at a local scale (i.e., the immediate context in which the stimulus is presented changes between conditions). In crowding, one usually contrasts visible and invisible conditions by manipulating the spacing or the similarity between the stimulus of interest and its flankers. This manipulation does not fulfill the criterion of constant stimulation, and therefore is not optimal in the context of a contrastive study of consciousness. However, by relying on psychophysical thresholding methods, it was shown that one can obtain conditions in which a crowded feature (i.e., the tilt of an oriented line) can be discriminated in some trials, but not in others, while the whole display remains constant (Faivre and Kouider, 2011a). Future studies are needed to assess whether such conditions can be obtained for multi-feature stimuli (e.g., faces).

As we mentioned above, “breaking CFS” is a popular variant of classical measures of after-effects (AE) which consists in measuring the time it takes for a stimulus to emerge into awareness despite suppression, and comparing possible differences between experimental conditions (Gayet et al., 2014; Jiang et al., 2007). Although this approach might potentially satisfy the criterion of constant stimulation, it suffers from methodological flaws, which are described in this volume [see the review by Stein and Sterzer (2014) and Gayet et al. (2014) in this research topic, as well as Stein et al., 2011]. Other studies have attempted to minimize the difference in terms of stimulation between conscious and non-conscious conditions, notably by presenting to the same eye the stimulus of interest superimposed to the mondrians in the conscious condition (e.g., Harris et al., 2011). It appears that reaching reliable perceptual changes at constant stimulation still requires efforts, both for crowding and CFS. Only then those two methods could be used for a strict contrastive description of the neural correlates of consciousness.

### IS IT REALLY INVISIBLE?

Crowding-induced undiscriminability and CFS-induced undetectability represent two qualitatively distinct perceptual impairments. It implies that crowding and CFS stem from different mechanisms that may be responsible for some of the specific features we describe hereafter. Although the extent of crowding varies depending on stimuli and participants, it is rather stable over time, which insures the possibility of prolonged experimental sessions and therefore accurate measures (but see Chung, 2007; Sun et al., 2010; Hussain et al., 2012 for learning effects during crowding occurring across daily experimental sessions). In CFS, a decrease of contrast detection thresholds after extensive training was recently documented (Ludwig et al., 2013). In addition, we noticed that a small proportion of observers (~5%) remain partially or completely immune to CFS: in some cases, binocular fusion seems absent, so that the stimulus of interest appears superimposed to the mondrians. In some other cases, suppression appears to break after shorter and shorter durations over an experimental session, which undermines the possibility of displaying long-lasting stimuli without awareness (this applies specifically to dynamic stimuli, see below). These observations are mainly based on personal experience, and inter-individual differences remain to be tested systematically for both methods. Likewise, no study has directly compared the causes of discrimination vs. detection impairments occurring respectively in crowding and CFS.

### HOW ECOLOGICAL IS IT?

As any sensory organs, the visual system is tuned according to the optical properties naturally present in the environment. Therefore, the contrast between conscious and non-conscious vision may be most accurate when performed in ecological conditions, which mimic the natural environment. As noted above, crowding is a natural phenomenon, occurring when exploring any visual scene composed of multiple objects appearing in the periphery of the visual field. By contrast, one rarely faces two rival stimuli presented to each eye, one of them being much more salient than the other as it is the case in CFS. In the next section, we will assess whether the apparent ecological superiority of crowding over CFS is reflected in terms of empirical results.

## LEVELS OF REPRESENTATION FOR NON-CONSCIOUS CONTENTS

In this part, we present the main findings obtained for stimuli rendered invisible with CFS and crowding, at different levels of visual processing: single features, motion signals, multi-feature objects (including faces), and semantic contents. By features, we mean discrete components of an image that are detected independently of each other (Suchow and Pelli, 2013). By semantic content, we refer to the meaning conveyed by a physical signal. We only review results that are based on indirect measures of non-conscious processing, whereby a modulation of neural or behavioral responsiveness follows the presentation of an invisible stimulus. Hence, we do not cover the literature based on the “breaking CFS” technique, whose potential confounds in terms of partial awareness are discussed in this research topic (see, Gayet et al., 2014; Stein and Sterzer, 2014).

### NON-CONSCIOUS PROCESSING OF SINGLE FEATURES

#### Low-level after-effects

Before the emergence of CFS about a decade ago, several studies showed that simple features rendered invisible by binocular rivalry produce specific sensory illusions called AE. The observation of such AE is considered as a behavioral signature of non-conscious processing, for simple features such as tilted lines (tilt AE, Wade and Wenderoth, 1978), square-wave gratings (spatial frequency AE, Blake and Fox, 1974), McCollough-type gratings (orientation-contingent color AE, White et al., 1978), and translational motion (motion AE, Lehmkuhle and Fox, 1975; O’Shea and Crassini, 1981).

Building upon those precursors, and under more reliable suppression conditions (Tsuchiya et al., 2006), similar results were found under CFS regarding tilt AE (Bahrami et al., 2008). Likewise, crowded tilted lines are also known to induce tilt AE (He et al., 1996), and priming (Montaser-Kouhsari and Rajimehr, 2005; Faivre and Kouider, 2011a). Yet, spatial frequency and orientation-contingent color AE remain to be tested under both CFS and crowding.

Brightness is another low-level feature whose perception under CFS has been studied using contextual illusions. On the one hand, it was shown that the simultaneous brightness contrast illusion (i.e., a gray circle appearing brighter against a dark background than against a light background) persists even when the spatial context inducing the illusion (the background region that surrounded two physically identical target circles) was suppressed from awareness by CFS (Harris et al., 2011). Yet, the same study reported that the Kanizsa triangle illusion (i.e., the illusion of a luminance contour induced by spatially distinct elements prompting the visual system to assume the presence of an occluding surface) did not persist when the inducer elements were suppressed from awareness^2^. These findings suggest that under CFS, the low-level processes involved in brightness perception (e.g., background luminance) can occur without awareness, while the higher-level ones involved in the assignation of surface borders through perceptual completion do not. Interestingly, positive evidence for the Kanizsa triangle illusion was found when the inducer elements were crowded (Lau and Cheung, 2012).

Over the years, a debate has emerged as to know whether low-level AE such as the tilt AE are stronger when induced by visible compared to invisible stimuli. He et al. (1996) first suggested that crowding had no influence on the amplitude of tilt AE induced by high-contrast gratings. On the other hand, it was shown that both tilt and motion AE were smaller when low-contrast stimuli were rendered invisible by binocular rivalry and crowding, compared to a situation of full visibility (Blake et al., 2006). This study further suggested that the independence between AE amplitudes and visibility found previously might stem from a saturation of the adaptive response at high stimulus contrast. Yet, a subsequent study showed that when manipulating carefully attentional amplification, low-contrast stimuli could induce the same AE whether crowded or not (Bi et al., 2009). Finally, evidence from fMRI indicated that crowding had a detrimental effect on AE amplitude at the level of V2/V3, but not at the level of V1 (where tilt AE are supposed to stem from, see Fang and He, 2008). Taken together, these results suggest that crowding may be seen as a bottleneck impacting visual processes differently depending on their position along the visual pathways.

If the independence between AE amplitude and crowding remains debated, the evidence is clearer for CFS: the very first paper introducing CFS showed that it had a detrimental effect on the amplitude of after-images (Tsuchiya and Koch, 2005), and recent findings using fMRI strengthened this idea by showing that CFS decreases the activity from signals as early as in the primary visual cortex (Watanabe et al., 2011; Yuval-Greenberg and Heeger, 2013). Comparing these results with those of Fang and He (2008) described above, one could deduce that crowding impacts visual processes at later stages compared to CFS. However, considering the high variability across experiments, we argue that clear conclusions should be drawn from systematic comparisons only (see discussion).

### NON-CONSCIOUS PROCESSING OF MOTION SIGNALS

As we mentioned in the introduction, CFS and crowding present the advantage of maintaining stimuli out of awareness for potentially long durations. So far, most studies that took advantage of this property focused on motion processing, by measuring motion AE from invisible stimuli. In CFS, translational motion has first been shown to induce motion AE that did not transfer across eyes, suggesting that the underlying processes are quite low-level (Maruya et al., 2008). Yet, with a slightly different setup, AE induced by translational and spiral motion were found to transfer across eyes, and to depend on attentional amplification (Kaunitz et al., 2011). Recently, AE from apparent and biological motions (i.e., point-light walkers) were found under CFS (Faivre and Koch, 2014b). However, it was found that the extent of temporal integration was smaller under CFS than in condition of full visibility (i.e., ~100 ms vs. ~1s for apparent motion, and ~1 vs. ~3s for biological motion, respectively). Taken together, these results suggest that motion processing is enabled under CFS at various levels of complexity, but with restricted periods of temporal integration. Interestingly, it was also shown that the processing of implied motion (i.e., static pictures depicting someone or something moving) was not enabled in the absence of awareness, suggesting that CFS has a detrimental effect not only on temporal but also spatial integrative processes (Faivre and Koch, 2014b).

Under crowding, translational motion is known to shift the apparent location of a subsequent stimulus (Whitney, 2005; Bressler and Whitney, 2006), while spiral motion (Aghdaee, 2005; Aghdaee and Zandvakili, 2005) and apparent motion (Rajimehr et al., 2004) also induce AE. In addition, crowded biological motion in the form of dynamic facial expressions were found to be processed despite crowding, notably through the dorsal visual pathway (Faivre et al., 2012b, see below). It appears from these findings that, as in CFS, motion processing at various levels of complexity is enabled under crowding.

### NON-CONSCIOUS PROCESSING OF MULTI-FEATURE OBJECTS

#### Face stimuli

Among all stimuli composed of multiple features, faces have undoubtedly triggered the most interest in the field of non-conscious vision, including studies relying on CFS and crowding. Beyond its obvious ecological value, a single face stimulus conveys multiple levels of information which allows for probing non-conscious processing at several levels of complexity. Here, we review the evidence for non-conscious face processing along two axes: the representation of facial identity and the emotional processing of facial expression.

Jiang and He (2006) were the first to focus on face processing under CFS with fMRI. They found that the fusiform face area was more activated by fearful or neutral faces compared to scramble faces. In addition, they found that the amygdala and superior temporal sulcus were more activated by fearful compared to neutral faces. Recently, it was found that this activation in the superior temporal sulcus was only present in participants with high negative affectivity (a dispositional trait relevant to psychopathology, see Vizueta et al., 2012). A subsequent study reported that category-specific responses induced by invisible faces vs. houses in fusiform and parahippocampal cortices can be only obtained when using multivariate pattern analysis, rather than univariate techniques, suggesting that the fine-scale pattern of activity within these areas encodes the features of invisible objects (Sterzer et al., 2008). Building upon these fMRI studies, the same research teams then focused on the electromagnetic correlates of non-conscious face processing. Jiang et al. (2009) found electroencephalographic responses to faces vs. scrambled faces in posterior occipital areas (between 140 ms and 200 ms after stimulus onset, arguably similar to the classical N170 component for face processing), followed by responses to fearful vs. neutral faces along superior temporal regions 220 ms after stimulus onset. Sterzer et al. (2009) confirmed the category-specific differences they found with fMRI in a magnetoencephalographic study, by documenting an M170 component in response to invisible faces vs. houses along the fusiform cortex. Taken together, these results based on hemodynamic and electromagnetic correlates of neural activity suggest that the signals conveying both face-specific information (i.e., face vs. scramble or fearful vs. neutral face) and category-specific information (i.e., face vs. house) are not abolished by CFS.

At the behavioral level, the evidence for processing of facial identity under CFS is less convincing. Using a method similar to CFS, Moradi et al. (2005) first attempted to measure identity AE, that is a bias for the perception of a specific facial shape after the observer is exposed to an adapting face that has opposite global features (its “antiface”). They found that such identity AE occurred when the adapting face was visible, but completely vanished when it was invisible. Later, Stein and Sterzer (2011) reported that identity AE could actually be induced by invisible faces, though with a reduced amplitude, and without interocular transfer (i.e., when the adaptation face and the target are presented to different eyes), suggesting that it probably stemmed from low-level processes. Similarly, Barbot and Kouider (2012) found that the identity of invisible faces induced repetition priming effects, but with no interocular transfer: participants were faster to categorize a target face as famous when it was preceded by an invisible identical vs. different prime face presented to the same eye. As primes and targets were of different sizes (i.e., 20% size difference), it was argued that these priming effects genuinely reflected non-conscious processing of facial identity. Yet, it was recently found that the similarity between a prime and a target differing only in terms of size could be captured as early as in the primary visual cortex, up to 70% of size difference (Faivre and Koch, 2014a). This raises the possibility that identity priming for faces reflects low-level overlap rather than the activation of face representations *per se*. In addition, the existence of identity repetition priming in conditions of complete unawareness was recently challenged by a study reporting that priming effects are indeed induced by faces whose identity is invisible, but critically, only when lower level features like color or location are visible (i.e., partial awareness, see Mudrik et al., 2013). We come back to the issue of partial awareness in the discussion. Overall, the behavioral evidence for the processing of facial identity remains inconclusive.

Now regarding emotional processing under CFS, an influential study first reported that observers’ attention could be attracted to or repelled from invisible erotic stimuli, depending on observer’s gender and sexual orientation (Jiang et al., 2006). Later, it was shown that invisible adaptors depicting facial expressions of anger, fearfulness and happiness could bias the way a subsequent target face was perceived (i.e., facial expression AE, see Adams et al., 2010). Contrary to what was observed for the processing of identity, these results cannot stem from low-level retinotopic similarities, as adaptor and target stimuli were presented to opposite sides of the visual field and to different eyes. These two studies reinforced the idea of a “special status” for emotional stimuli, as objects that are processed without awareness notably along subcortical routes (Tamietto and de Gelder, 2010). Yet, facial expression AE were subsequently dismissed on the basis that it probably stemmed from residual visibility and attentional confounds (Yang et al., 2010; see Adams et al., 2011 for a response). Supporting this idea, another study showed that aftereffects from gender and race information were absent under strict control of awareness (Amihai et al., 2011). New elements to this debate were recently added by two studies, in which invisible fearful faces were found to change skin-conductance responses, both in the context of fear conditioning (Raio et al., 2012) and preference judgments (Lapate et al., 2013). In addition, preference judgments biases were found to be induced by angry – but not happy – faces rendered invisible by CFS (Almeida et al., 2013, but see Faivre et al., 2012a for negative results under masking and CFS; de Zilva et al., 2013 for negative results about the mere exposure effect under CFS). We can conclude from this group of recent studies that unlike facial identity, facial expressions rendered invisible by CFS elicit responses that can be captured at the behavioral level^3^.

Under crowding, Faivre and Kouider (2011b) showed repetition priming of facial identity when the prime and target were presented 15° away from each other, suggesting that unlike what was shown for CFS (Barbot and Kouider, 2012), identity processing under crowding does not depend on retinotopic similarity. Furthermore, we showed that crowded facial expressions can bias subsequent affective judgments of neutral pictographs (happy faces elicited more pleasant judgments than angry faces, see Kouider et al., 2011). Moreover, the preference bias induced by crowding faces was not only induced by static (i.e., pictures) but also by dynamic (i.e., videos) facial expressions. Using fMRI coupled with univariate analysis, it was found that compared to a neutral face, static happy faces activated primarily the ventral visual pathway including the fusiform face area, which was functionally connected to the amygdala (Faivre et al., 2012b). By contrast, dynamic happy faces triggered the dorsal visual pathway (including the posterior parietal cortex) and the substantia innominata, a structure contiguous with the dorsal amygdala. To our knowledge, no multivariate pattern analysis has been applied to try to decode the content of crowded stimuli. Along the same lines, it was shown that crowded emotional faces could influence a conscious judgment (assessing the average emotion resulting of six flanker faces and one target crowded face) while the same inverted and scrambled faces could not (Haberman and Whitney, 2007, 2009). Like our results, this finding shows that despite preventing object recognition, crowding does not impede the processing of emotional information extracted from objects.

#### Tool stimuli and the dorsal visual stream

Besides faces, the processing of tools under CFS has also received much attention. Using fMRI, Fang and He (2005) first revealed that suppressed pictures of tools specifically activate the dorsal visual pathway, which is thought to support the guidance of actions (Goodale and Milner, 1992). At the behavioral level, it was shown that suppressed pictures of tools – but not of non-manipulable objects like animals – could facilitate the categorization of subsequent targets (i.e., categorical priming, see Almeida et al., 2008, 2010), suggesting that non-conscious processing in the dorsal – but not ventral – visual pathway can be used for recognizing manipulable objects. Recently, this finding was challenged by a study revealing that similar priming effects could actually be induced by any kind of elongated objects, rather than specifically manipulable objects (Hebart and Hesselmann, 2012; Sakuraba et al., 2012). As the previous priming effects may stem from such low-level confounds, the level of processing undergone by invisible tool stimuli remains unclear. Relying on multivariate analysis of blood-oxygen-level dependent signal, Hesselmann and Malach (2011) showed that features from invisible tools were encoded in the lateral occipital cortex, which rules out the possibility that stimulus energy was so low that all high-level processes were abolished. Most importantly, they also found that CFS equally reduced brain activity in the ventral and dorsal visual pathways, which challenges the original claim that CFS has no or little influence on the dorsal visual pathway (Almeida et al., 2008). The claim that CFS disrupts the ventral but not the dorsal visual pathway is further debated, as one study documented the capacity to grasp visually suppressed stimuli (Roseboom and Arnold, 2011, but see Ludwig et al., 2013; see also the comment on the study by Roseboom and Arnold, 2011 from the same authors).

Regarding crowding, although several lines of evidence suggest that multi-feature objects like arrows (Faivre and Kouider, 2011b), sequences of geometric shapes (Atas et al., 2013), or naturalistic objects (Fischer and Whitney, 2011) are processed in the absence of conscious discrimination, no study has investigated directly the processing of crowded tools. Yet, it was found that crowding decreases to the same extent the spatial resolution of both visually guided reaching and perception, suggesting that it impacts both the ventral and the dorsal visual pathways (Bulakowski et al., 2009).

### NON-CONSCIOUS PROCESSING AT THE SEMANTIC LEVEL

As of today, it remains unclear whether stimuli rendered invisible by CFS can be processed up to the semantic level. Combining the semantic-priming procedure with binocular rivalry, Zimba and Blake (1983) first presented prime words to an eye during either dominance or suppression phases of binocular rivalry. A semantic-priming effect (here on response times in a lexical decision task) was observed only when prime words were presented during dominance phases, suggesting that semantic processing is disabled during suppression phases.

Kang et al. (2011) extended this work by combining the semantic-priming procedure with CFS, and using the N400 component of human event-related potentials (ERPs) as an electrophysiological index of semantic processing. Here, an invisible target word (e.g., apple) was preceded by a semantically related (e.g., orange) vs. unrelated (e.g., doctor) prime word. Although target words usually elicit N400 components of smaller amplitudes when preceded by semantically related vs. unrelated primes, no such modulation was observed when the target was rendered invisible by CFS. As in binocular rivalry, this result lead the authors to conclude that semantic processing of words was disabled under CFS (but see Heyman and Moors, 2012, for possible theoretical and methodological issues).

Contradicting this negative result, it was recently shown that complex – rather than single – semantic stimuli (e.g., multiple-word phrases, basic equations) rendered invisible by CFS for longer durations (i.e., up to 2 s) can still be processed (Sklar et al., 2012). For instance, the result of an invisible equation (e.g., 9 – 3 – 4 = ) was found to facilitate the response to a subsequent target number congruent to the equation’s solution. This suggests that the equation had been non-consciously solved by the time the target appeared. In line with this study, Zabelina et al. (2013) investigated the semantic processing of triplets of words under CFS. In each trial, participants had to solve a compound remote associate problem, that is finding a word (e.g., apple) common to three seemingly unrelated words that were suppressed for seconds before being fully visible (e.g., pine, crab, sauce). Participants solved word problems faster following suppressed problem words than following suppressed irrelevant words. Interestingly, this priming effect was observed only when participants reached the solution by analysis rather than by insight, which led the authors to suggest that semantic processing but not semantic integration of the word triads occurred non-consciously. Here, however, since there was no physical difference between primes and targets (i.e., the task was performed once the triplet words became visible), priming may have stemmed from a perceptual rather than semantic facilitation. Indeed, participants may have processed the triplet words at a perceptual but not semantic level, which nevertheless would facilitate responses on the triplet words when they become visible.

In the same vein, using a setting in which participants heard a verbal label before performing a simple detection task wherein stimuli were pictures of familiar objects rendered invisible by CFS, Lupyan and Ward (2013) found that valid labels (words semantically related to the object) improved performance while invalid labels decreased performance. Yet, they also reported that the effectiveness of labels varied as a function of the match between the shape of the stimulus and the shape denoted by the label, suggesting that labels facilitated the perceptual processing of the suppressed objects rather than their semantic processing^4^.

Finally, two studies have investigated whether crowded stimuli can be processed at the semantic level. Yeh et al. (2012) showed that crowded single-character Chinese words were able to induce behavioral semantic-priming effects in a lexical decision task, with an effect amplitude similar to those induced by visible Chinese words. Recently, Peng et al. (2013) combined the semantic-priming paradigm with crowding while recording ERPs. As in the CFS study by Kang et al. (2011) described above, crowding was applied to target rather than prime words. Here, participants were required to judge whether the prime and target words were semantically related or not. Semantic priming was reflected both in terms of reaction times and in the amplitude of the N400 component, although effects were of smaller amplitudes for crowded compared to uncrowded targets. However, one should note that the discriminability of crowded targets was slightly above chance-level, which questions the non-conscious origin of these effects. Interestingly though, the authors report that long-lasting presentation of crowded targets is required in order to observe semantic priming, which suggests that sustained invisibility is beneficial when probing high-level processes.

### SUMMARY: WHAT CAN BE SAID OVERALL?

We here summarize what emerges from the sum of empirical results describing the depth of non-conscious processing under crowding and CFS over the last decade. First, as a tool to study non-conscious vision, it appears that the use of CFS is much more widespread than that of crowding (i.e., we numbered 50 vs. 21 studies addressing directly non-conscious processing with CFS vs. crowding). Overall, there is strong behavioral evidence for the processing of simple stimuli under both CFS and crowding, including luminance and contrast (CFS: Harris et al., 2011; crowding: Lau and Cheung, 2012), orientation (CFS: Bahrami et al., 2008; crowding: He et al., 1996; Faivre and Kouider, 2011b), and motion (CFS: e.g., Maruya et al., 2008; Kaunitz et al., 2011; Faivre and Koch, 2014b; crowding: e.g., Rajimehr et al., 2004; Whitney, 2005). Efforts remain to be made regarding the respective impact of crowding and CFS on the amplitude of such low-level processes (e.g., size of AE). Regarding multi-feature objects, the literature on CFS is rather controversial. As of today, no compelling behavioral evidence supports the processing of facial identity (e.g., Moradi et al., 2005; Stein and Sterzer, 2011), even though signals conveying facial identity may be detectable at the neural level, especially when using more subtle analyses such as multivariate pattern classification (Sterzer et al., 2008). By contrast, emotional stimuli like facial expressions seem to trigger both behavioral and neural responses (e.g., Adams et al., 2010). This discrepancy between the processing of facial identity and facial expressions suggests that the latter may be processed along subcortical routes that are not fully disrupted by CFS (Tamietto and de Gelder, 2010). One can conclude that behavioral measures (i.e., priming, AE) may not be suited for detecting the weak traces left by complex stimuli rendered invisible by CFS. Yet, physiological measures like skin conductance, electromagnetic or hemodynamic responses (associated with multivariate analysis) seem to indicate that CFS does not abolish the processing of complex stimuli such as faces (facial expressions, see Raio et al., 2012 or facial identity, see Sterzer et al., 2008) or tools (Hesselmann and Malach, 2011). Similarly, two studies (Sklar et al., 2012; Zabelina et al., 2013) seem to indicate that the processing of combination of words or numbers are processed up to the semantic level, although potential low-level confounds remain to be ruled out.

Compared to CFS, the literature on crowding is more limited, but also more consistent: all studies we found report positive behavioral results for the encoding of crowded multi-feature objects, including symbols, facial identity (Faivre and Kouider, 2011b), and facial expressions (Kouider et al., 2011), up to the extraction of semantic information (Yeh et al., 2012; Peng et al., 2013; although full indiscriminability of crowded stimuli is not always warranted, see above). It is unfortunate that compared to CFS, the neural basis of non-conscious processing under crowding remains largely uncovered (see Chicherov et al., 2014 for a recent study on the neural correlate of crowding).

From low-level features to semantic content, looking exclusively at the positive results we reviewed would lead to the conclusion that virtually any kind of visual process is enabled under crowding or CFS. One could derive from this observation that stimulus awareness has no functional role during visual processing (e.g., Hassin, 2013). Yet, a large portion of the results we reviewed are far from being unequivocal. Indeed, the literature on each specific topic often includes conditions of residual awareness, negative findings which are difficult to interpret, replication failures which most likely exist but remain undocumented, or inadequate conclusions due to experimental confounds (e.g., arguing for the processing of tools vs. elongated objects, or for semantic rather than perceptual processing, see above). Hence, this heterogeneous set of studies makes the whole picture of non-conscious vision under crowding and CFS difficult to interpret. Below, we discuss some potential reasons for this difficulty, and humbly propose tentative guidelines to manage this tremendously challenging task.

#### Is one better than the other?

**Tables 1** and **2** summarize what can be said regarding the respective advantages of crowding vs. CFS. Considering the lack of systematic methodological comparison, the criteria for choosing one or the other method mostly pertain to practical considerations. As mentioned in the introduction, if one’s aim is to study the processing of simple stimuli in the complete absence of awareness (e.g., an oriented line remaining undetectable), CFS would most likely do the trick. Crowding, on the other hand, is not as potent with single feature stimuli, and only impedes stimulus discrimination. Yet, if one’s aim is to measure the processing of complex objects in a natural environment (e.g., under conditions of virtual reality, see Pizzi et al., 2012), crowding seems like a valuable alternative to CFS.

**Table 1:** Summary of the comparison of crowding and CFS on different psychophysical features.

	Crowding	Continuous flash suppression
Stimulation	Binocular, peripheral	Monocular, foveal
Maximal duration	Unlimited?	~30s
Visibility impairment	Discrimination	Detection
Adjustable parameters	Eccentricity, flankers, contrast	Mondrians, contrast
Subjective change at constant stimulation	Only for tilted lines	Only with bCFS
Conditions of partial awareness	Not documented	Location, form, color
Sensitivity to attentional amplification	Yes	Yes
Efficacy for dynamic stimuli	Good	Good
Efficacy for faces	Good	Good
Efficacy for single features	Poor	Good
Robustness across subjects	Good	Fair
Compatibility with physiological measures	Good	Good

**Table 2:** Summary of the comparison of crowding and CFS on the level of evidence regarding non-conscious processing of various contents.

	Crowding	Continuous flash suppression
**Single features**
*Tilt AE*	Good, decrease of AE amplitude debated	Good, decrease of AE amplitude
Motion signals	Good	Good (decrease of temporal integration)
**Faces**
*Facial identity*	Good	Fair (results are mixed at the behavioral level)
*Facial expression*	Good	Good
Tools	Lack of studies	Fair (potential low-level confounds)
Semantic	Fair (few studies, potential visibility confounds)	Fair (potential perceptual confounds)

The main advantage of both crowding and CFS over the arsenal of techniques available in the field (Kim and Blake, 2005) is the possibility for sustained invisibility: a stimulus can be presented for several seconds, either in the form of a static picture or dynamic movie, while the observer accesses none or some of its features (see above). This improvement opens many research questions: how do conscious vs. non-conscious processes unfold over time? Can temporal structures spread over long durations be integrated into unique representations? For what kind of processing is sustained invisibility beneficial? One obvious case is the processing of dynamic stimuli (e.g., motion). Another one is the processing of long-lasting stimuli inducing neural fatigue (e.g., inducing tilt AE). Displaying static stimuli for long durations is known to potentially overstimulate the visual system, and transform facilitatory into inhibitory effects (see Faivre and Kouider, 2011a; Barbot and Kouider, 2012). Besides this phenomenon, several studies investigating high-level cognitive processes mentioned the benefit of using long-lasting static stimuli (i.e., several hundred of milliseconds up to 3 s) as it gives them the time necessary for elaborate processing. For instance Bahrami et al. (2010) had invisible gabor patches conveying numerosity information presented for durations up to 3 s. Sklar et al. (2012) had sentences and equations suppressed from awareness for up to 2 s. It would be interesting to test whether such non-conscious processes under crowding and CFS are enabled when shorter stimuli are used. In crowding, Peng et al. (2013) explicitly mentioned the presence of semantic priming when crowded words were presented for 1 s but not 350 ms. This is at odds with the numerous results showing that processing of words or digits presented for a few 10s of milliseconds are enabled despite masking (see Kouider and Dehaene, 2007 for a review). In those conditions, determining what is left to crowding and CFS compared to masking or other techniques requires systematic comparisons.

## CROWDING AND CFS: WHAT’S NEXT?

Altogether, the numerous studies reviewed above provide a rather unclear and incomplete picture of the nature of non-conscious vision under crowding and CFS. This, in our opinion, is due to three main methodological limitations (see also Yang et al., current issue, for a standardized approach in CFS).

The first methodological limitation is that most studies rely on “all-or-none” designs, whereby only the presence vs. the absence of an non-conscious process is assessed. If enabled, this process typically gives rise to a measurable effect (e.g., priming, AE, changes of neural activity, etc.), while if it is disabled, a null effect is observed. Such null effects can be hardly interpretable, and are in fact rarely published, giving rise to a bias in the literature on non-conscious vision (i.e., file drawer effect, see Rosenthal, 1979). Thus, rather than “all-or-none” designs, we argue that the field would benefit from the use of parametric designs. First, parametrization can be applied to the stimulus visibility, in order to compare a process at distinct levels of awareness (e.g., with different degrees of crowding or CFS). This allows for estimating quantitatively the role of stimulus visibility for a given process in terms of effects’ amplitude (e.g., the amplitude of tilt and motion AE at various levels of crowding and binocular rivalry, see Blake et al., 2006), effects’ dynamics (e.g., the time it takes for an effect to arise over the experimental session) or effects’ robustness (e.g., how sensitive an effect is to attentional manipulations). Secondly, parametrization can be applied to the stimulus complexity, in order to compare conscious and non-conscious processes at distinct levels of representation (e.g., probing facial identity and expression with the same stimuli). In this context, the observation of a null effect obtained at one level of representation may be confirmed by the presence of a positive effect showing that the stimulus is nevertheless processed at a lower level of representation (e.g., evidence for lexical but not semantic processing). Accordingly, one would be able to estimate the impact of crowding and CFS at distinct levels along the visual pathways for a single stimulus, as it was tentatively done in binocular rivalry (Nguyen et al., 2003). Not only this strategy would help probing the limits of non-conscious vision more systematically, but also lead to a better understanding of the mechanisms at the origin of invisibility under crowding and CFS.

The second methodological limitation is that most studies usually measure non-conscious processing relying on a single technique to prevent stimulus awareness (but see Blake et al., 2006; Almeida et al., 2008, 2013; Kanai et al., 2010; Faivre et al., 2012a; Stein et al., 2013, for examples of studies measuring a process under different techniques). Considering that each study uses different experimental setups (i.e., in terms of stimulus set, hardware, indirect measure of processing, etc.), it is difficult to conclude about the superiority of one technique over the other. Yet, if one wants to describe the limits of non-conscious vision, one has to disentangle what can be attributed to the method employed to render the stimulus invisible (i.e., stimulus duration, contrast, position in the visual field, etc.) and the limits attributed to the nature of non-conscious processing *per se*. This may require performing studies at larger scales, in which the dependencies between one specific process and visual awareness are assessed with a set of complementary methods and a single stimulus set.

The third methodological limitation concerns measures of stimulus awareness. Although some efforts are made at the theoretical level to reach a consensus regarding a definition for stimulus awareness (e.g., Seth et al., 2008; Sandberg et al., 2010), most studies diverge in their use of visibility measures. Objective measures include detection tasks (i.e., determinate if the stimulus is present or not), discrimination tasks (i.e., recognize a stimulus from its scrambled version), or categorization tasks (i.e., distinguish two stimuli from different categories). Invisibility is usually taken as granted from chance-level performance in any of these measures. Yet, each of them clearly implies a different definition of invisibility. For instance, performance on a detection task in a crowding experiment would be clearly above chance, as only the discriminability of a crowded stimulus is impaired (Levi, 2008). Moreover, long periods of partial awareness are described under CFS, in which observers have access to specific features of a stimulus like its color or location, but not others like its orientation (Hong and Blake, 2009; Zadbood et al., 2011). In this situation, observers are likely to perform at chance-level in one but not the other objective visibility task. The lack of consistency in the assessment of stimulus awareness is particularly problematic considering that these situations of partial awareness are known to potentially drive supposedly non-conscious effects (Kouider and Dupoux, 2004; Kouider et al., 2010; Mudrik et al., 2013). In order to refine the level of awareness associated with one or the other technique, objective measures may be used in synergy with subjective ones using either continuous (e.g., Sergent and Dehaene, 2004) or discrete scales (Ramsøy and Overgaard, 2004). Finally, each measure may be performed at the single trial level, in order to account for training or fatigue effects^5^. This is particularly relevant in case stimuli are presented for long periods of time during which awareness may fluctuate.

## Conflict of Interest Statement

The authors declare that the research was conducted in the absence of any commercial or financial relationships that could be construed as a potential conflict of interest.

## Footnotes

1 Discrimination with ~75% accuracy was obtained at low eccentricities for single features like orientation (2.5° in Parkes et al., 2001), size, saturation, and hue (2–15° in van den Berg et al., 2007). Further research is needed in order to establish whether crowding differs between single and multi-feature stimuli.

2 See Wang et al. (2012) for positive results based on bCFS.

3 Interestingly, much more positive results were found using the breaking CFS method (Jiang et al., 2007; Yang et al., 2007; Tsuchiya et al., 2009; Zhou et al., 2010; Stein et al., 2012, 2013; Stewart et al., 2012; Gray et al., 2013). These results and the apparent discrepancy with the ones we reviewed are discussed in this research topic (see Gayet et al., 2014; Stein and Sterzer, 2014).

4 As in for what we noted regarding face processing, much more evidence of semantic processing was obtained using bCFS (Jiang et al., 2007; Costello et al., 2009; Mudrik et al., 2011; Yang and Yeh, 2011; Sklar et al., 2012).

5 One can also combine these two measures in a multiple-alternative forced choice task, in which each alternative corresponds to one objective response associated with a subjective level of visibility (for instance, letters of different sizes in which the letter type indicates the category of a stimulus, and its size indicates the confidence with which the decision is made).
